# Leveraging National Cancer Institute Programmatic Collaboration for Single Radiopharmaceutical Drug Master Files

**DOI:** 10.3389/fonc.2019.00573

**Published:** 2019-06-28

**Authors:** Charles A. Kunos, Jacek Capala, Susan Percy Ivy

**Affiliations:** ^1^Cancer Therapy Evaluation Program, National Cancer Institute, Bethesda, MD, United States; ^2^Radiation Research Program, National Cancer Institute, Bethesda, MD, United States

**Keywords:** radiopharmaceutical, drug master file, targeted radioisotope therapy, targeted radiation therapy, cancer, National Cancer Institute (NCI)

## Abstract

Targeted radiopharmaceutical conjugates intended for therapeutic use often are made of three key components, a decaying radionuclide, a chemical chelator/linker, and a targeted molecular entity. The National Cancer Institute (NCI) Experimental Therapeutics Program has accepted four radiopharmaceutical drug products so far that fit the targeted radiopharmaceutical conjugate class. As the NCI sharpens its thinking about its role as an investigational new drug sponsor for radiopharmaceuticals in clinical development, it has considered the relative merits of modular radiopharmaceutical drug master files. Here, the NCI provides its perspective on modular radiopharmaceutical drug master files as it initiates a clinical development program for such agents and further organizes its radiopharmaceutical Small Business Innovation Research portfolio.

## Introduction

A Drug Master File (DMF) is an electronic format submission to the United States (U.S.) Food and Drug Administration (FDA) that might provide confidential and detailed data about the facilities, the methods, or the chemical/molecular entities used in the manufacture, processing, packaging, handling, or storage of one or more human drug substances or drug products (1).

Under U.S. Federal law [21 CFR Part 312], the FDA evaluates the source and preparation of a new drug product intended for human use under an Investigational New Drug Application (IND). An IND is the method through which a human clinical trial sponsor obtains an exemption from the FDA to distribute a new drug product across state lines prior to an approved marketing application (2). Also, when a New Drug Application (or NDA) is submitted for FDA review, a description of the methods used in the synthesis of a new drug substance is required under U.S. Federal law [21 CFR 314.50(d) (1) (i)] (3). A DMF provides the FDA a confidential mechanism to review the details of the chemistry, manufacture, and controls for a particular drug product without disclosure to other parties engaged in the human clinical development of the drug product. The DMF is considered an important and relevant mechanism because the manufacture of a drug product, whether by a process of synthesis, fermentation, or isolation, often begins with relatively impure materials. Later production steps involve the preparation or the transformation of intermediates, that have themselves undergone a characterization and purification, that permit final new drug product formulation. It is felt that quality and purity of a new drug product cannot be guaranteed only by end-of-the-line product validation. Rather, it is advocated that quality and purity of final drug formulations depend upon appropriate and controlled manufacture throughout the process. This means (a) appropriate quality and purity of the starting materials, reactants, and reagents; (b) determination and utilization of in-process controls for any intermediates; (c) consistency and adherence to validated processes; and (d) accuracy of the final release tests of the new drug product.

For this article, the National Cancer Institute (NCI) provides its perspective on modular radiopharmaceutical drug master files as it initiates a clinical development program for radiopharmaceuticals in new cancer disease indications. The NCI is in the process of further organizing its radiopharmaceutical Small Business Innovation Research (SBIR) portfolio of 19 next-generation radiopharmaceutical cancer technology projects invested in between 2015 and 2017. As the NCI seeks a return on its radiopharmaceutical SBIR investment and begins to push brand-new radiopharmaceutical therapies to patients in clinical trials (4), it is important to discuss the value added for modular radiopharmaceutical DMFs to speed clinical development of radiopharmaceutical new molecule entities.

## Challenges and Opportunities

**Figure 1:**
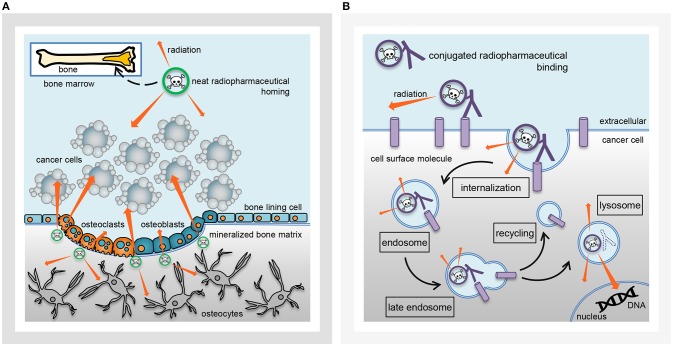
Radiopharmaceutical classification. Charted are key radiobiological aspects of radiopharmaceuticals of the **(A)** neat class (i.e., no targeting ligand) or **(B)** conjugated class [i.e., has targeting ligand (protein or antibody)]. **(A)** A neat radiopharmaceutical might act as a mineral mimetic [such as calcium in the case of radium-223 (green radionuclide)], home in on areas of tumor-related bone turnover in affected bones, incorporate and lock into mineralized bone matrix, and lethally irradiate nearby tumor cells for tumor control, as well as, osteoclasts and osteoblasts so as to mitigate abnormal tumor-related bone formation. **(B)** A conjugated radiopharmaceutical with a targeting ligand [like thorium-227 antibody conjugates (purple radionuclide)] might bind to cancer cell surface molecules, internalize as part of endosomes, and lethally irradiate nuclear DNA from within the cancer cell.

**Figure 2:**
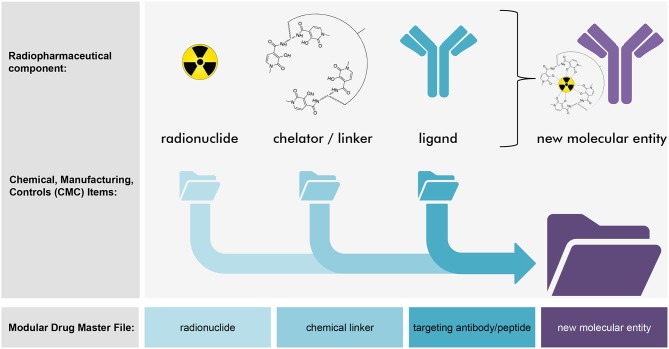
Strategy for modular radiopharmaceutical drug master files. Depicted are NCI's thoughts on a conceptual strategy for modular radiopharmaceutical drug master files (DMFs). This illustration describes a radiopharmaceutical from the conjugate class. Here, the new molecular entity is made up of three components—a radionuclide, a chemical chelator or linker, and a targeting ligand (such as the depicted antibody). Each component might have its own individual DMF that details its individual chemistry, manufacturing, and controls. By cross-reference, a modular radiopharmaceutical DMF might detail the new molecular entity's chemistry, manufacturing, and controls without duplicating forms, filings, and effort. In concept, this approach speeds clinical development of a radiopharmaceutical that might be considered a new molecular entity by regulatory agencies.

Radiopharmaceutical formulations intended for the oncology patient clinic might be inhaled, ingested, instilled, or infused by vein. These radioactive drugs strive for precise and accurate molecular conveyance of energy-rich radiation to cancer cells residing in tumors or circulating in the blood or marrow. The classification of radiopharmaceuticals has followed two naming conventions (Figure 1)—neat (which means that the radiopharmaceutical lacks a targeting ligand), or, conjugated (which indicates the radiopharmaceutical has a targeting ligand). Radium-223 dichloride is a neat radiopharmaceutical in that when it is administered intravenously as a slow bolus injection, it homes in to foci of bone turnover as a calcium mimetic without the aid of a targeting ligand. Thorium-227 (the parent radionuclide of radium-223) acts in a different way as a conjugated radiopharmaceutical. A conjugated radiopharmaceutical may have three molecular entities—a radioactive payload, a linker, and a targeting ligand (Figure 2). Each component in its own way possibly contributes to the human safety profile of the entire new molecular entity. For this reason, NCI has adopted the approach for conjugated radiopharmaceuticals to consider the safety of radionuclide, its cleaved/non-cleaved linker, and “cold” radiopharmaceutical targeting molecular entity prior to implementing early phase human clinical trials of the radiopharmaceutical.

There are challenges and opportunities when it comes to the submission of a radiopharmaceutical DMF. First, a DMF is not required by law or FDA regulation, but their provision is in 21 CFR 314.420. Thus, a DMF submission is considered at the sole discretion of the radiopharmaceutical holder. There are positive opportunities linked with the submission of a radiopharmaceutical DMF in that a single DMF may be used repeatedly to support an IND, NDA, Abbreviated NDA (ANDA), another DMF, and an Export Application, or amendments or supplements to any of these entities. However, the FDA is very careful to recognize that a DMF is not a substitute for any of these entities. Second, a DMF is not sanctioned or refused—it is a mechanism that permits the FDA to review the “closed” and confidential technical aspects of the chemistry, manufacture, and controls for a particular drug product without disclosure to other parties. Third, a DMF is reviewed only when there is an intentional review of an IND, NDA, ANDA, or an Export Application. When an FDA applicant references its own DMF, the applicant refers the FDA to up-to-date chemistry, manufacture, and controls directly rather than creating a new DMF that might duplicate many subcomponents. FDA guidelines for DMFs do not impose mandatory elements of the file [21 CFR 10.90(b)]; FDA guidelines do, however, offer guidance on suitable tactics to meet regulatory requirements.

## Perspectives On Radiopharmaceutical Drug Master Files

In the current FDA review process, certain new drugs are classified as new molecular entities (“NMEs”). Many radiopharmaceuticals contain active components that have not been approved for human use by the FDA before, either as a single ingredient drug or as part of a combination drug product formulation. In the current state of affairs, a radiopharmaceutical formulation might just be labeled an NME for administrative purposes, but nonetheless contain active components that have previously been approved by FDA when used in other drug formulations. Thus, there arises a regulatory conundrum of deciding whether a radiopharmaceutical NME can draw upon established DMFs or whether new DMFs need filed.

From the NCI's perspective, there are four DMF types. A Type II DMF contains information and supporting data for drug product, drug substance, drug substance intermediate, or material used in their preparation. This DMF type is often limited in scope to a single information and data set for drug product, substance, intermediate, or material. A Type III DMF contains packaging material information and supporting data. A Type IV DMF contains information and supporting data on any excipient, colorant, flavor, essence, or material used in this aspect of preparation. A Type V DMF lists accepted reference information not included in the other types. The archived and no longer accepted Type I DMF listed manufacturing site, facilities, operating procedures and personnel information that was not specific to a drug substance. One important perspective from the viewpoint of the NCI is the notion that any DMF can be cross-referenced to any other DMF, reducing duplicity and speeding review.

A radiopharmaceutical like radium-223 might submit all of the technical aspects of its chemistry, manufacture, and controls for drug formulation under its NDA (5).

A radiopharmaceutical might also use references to multiple DMFs. In the example of a conjugated radiopharmaceutical that is made up of an alpha-particle emitting radionuclide, a chelator with chemical linker, and targeting molecular entity like a ligand antibody, a modular DMF might cross-reference the DMF of each component (Figure 2). Each radiopharmaceutical component would have a registered DMF with the FDA that summarizes all substantial steps in the individual manufacture and control of drug intermediate or substance production. In this scenario, the use of cross-reference letters to each of the three DMFs and a master DMF for the new molecular entity radiopharmaceutical would support a submitted IND, NDA, ANDA, or Export Application.

Another advantage of a modular DMF for radiopharmaceuticals lies in the ease with which new IND, NDA, ANDA, or Export Applications could be reviewed when a radionuclide is the only component switched for a NME. For instance, it may be advantageous to swap out a therapeutic alpha-particle emitting radionuclide for a desired positron-emitting radionuclide for diagnostic imaging. Here, all of the chelator/linker and targeting entity components are the same and cross-reference letters to existing DMFs are used to support a new application. Such a solution reduces duplication and government resource waste. In another example, a switch of ligand antibody for a ligand bicycle protein permits yet another NME DMF to be created from already existing information and supporting data. Only those items previously undescribed in existing DMFs need DMF submission. Thus, one could envision a series of overlapping cross reference letters that draw components of other DMFs together so that investigators and pharmaceutical collaborators do not waste effort on filing already established and approved chemistry, manufacturing, and control processes. This ensures safety and speeds innovative development of agents for patients.

## Conclusion

In summary, radiopharmaceutical DMFs are not required by U.S. Federal law or FDA regulation, but the data within detailing the chemistry, manufacture, and control of drug products are required when an IND, NDA, or ANDA is intended. In this article, the NCI's perspective on a modular radiopharmaceutical DMF aligns well with the current thinking of the FDA. The modular DMF reduces duplicity by breaking down individual components of a radiopharmaceutical, cross-references DMFs for each individual component, and then files a master DMF for the final radiopharmaceutical formulation to speed regulatory aspects of NME clinical development.

## Data Availability

No datasets were generated or analyzed for this study.

## Ethics Statement

The research presented in this article involved the collection or study of existing data, documents, and records that were publicly available. The research is regarded exempt from Institutional Review Board oversight.

## Author Contributions

CK, JC, and SI contributed to the collection and review of any perspective data, analysis, authentication, writing, and approval of this manuscript. The views expressed are those of the authors and not those of the U.S. Federal government. Links or discussion of specific radiopharmaceutical drug products do not constitute endorsement.

### Conflict of Interest Statement

CK, JC, and SI declare that the research was conducted in the absence of any commercial or financial relationships that could be construed as a potential conflict of interest.

## References

[1] U.S. Food and Drug Administration Drug Master Files (DMFs). Available online at: https://www.fda.gov/drugs (accessed December 6, 2018).

[2] U.S. Food and Drug Administration Investigational New Drug (IND). Available online at: https://www.fda.gov/drugs (accessed December 6, 2018).

[3] U.S. Food and Drug Administration. New Drug Application (NDA). Available online at: https://www.fda.gov/drugs (accessed December 6, 2018).

[4] Kunos CA Capala J. National Cancer Institute Programmatic Collaboration for Investigational Radiopharmaceuticals. Am Soc Clin Oncol Educ Book. (2018) 38:488–94. 10.1200/EDBK_200199 30231365

[5] U.S. Food and Drug Administration Xofigo (radium Ra 223 dichloride) Injection, for Intravenous Use. Available online at: https://www.accessdata.fda.gov/drugsatfda__docs/label/2013/203971lbl.pdf (accessed December 6, 2018).

